# Transcriptome-based biomarker prediction for Parkinson’s disease using genome-scale metabolic modeling

**DOI:** 10.1038/s41598-023-51034-y

**Published:** 2024-01-05

**Authors:** Ecehan Abdik, Tunahan Çakır

**Affiliations:** Department of Bioengineering, Gebze Technical University, Kocaeli, Turkey

**Keywords:** Computational biology and bioinformatics, Molecular biology, Systems biology, Biomarkers, Parkinson's disease

## Abstract

Parkinson's disease (PD) is the second most common neurodegenerative disease in the world. Identification of PD biomarkers is crucial for early diagnosis and to develop target-based therapeutic agents. Integrative analysis of genome-scale metabolic models (GEMs) and omics data provides a computational approach for the prediction of metabolite biomarkers. Here, we applied the TIMBR (Transcriptionally Inferred Metabolic Biomarker Response) algorithm and two modified versions of TIMBR to investigate potential metabolite biomarkers for PD. To this end, we mapped thirteen post-mortem PD transcriptome datasets from the substantia nigra region onto Human-GEM. We considered a metabolite as a candidate biomarker if its production was predicted to be more efficient by a TIMBR-family algorithm in control or PD case for the majority of the datasets. Different metrics based on well-known PD-related metabolite alterations, PD-associated pathways, and a list of 25 high-confidence PD metabolite biomarkers compiled from the literature were used to compare the prediction performance of the three algorithms tested. The modified algorithm with the highest prediction power based on the metrics was called TAMBOOR, TrAnscriptome-based Metabolite Biomarkers by On–Off Reactions, which was introduced for the first time in this study. TAMBOOR performed better in terms of capturing well-known pathway alterations and metabolite secretion changes in PD. Therefore, our tool has a strong potential to be used for the prediction of novel diagnostic biomarkers for human diseases.

## Introduction

Parkinson’s disease (PD) is the most common neurodegenerative motor function disorder, which affects 0.1% of the population older than 40 years^1^. This ratio reaches 2–3% for the population older than 65^2^. The world’s population is aging as a result of the improvements in health care and life quality, causing an increase in the PD incidence rate. The number of people with PD is expected to double between 2015 and 2040^3^. The current treatments mainly relieve symptoms, but there is no neuroprotective treatment yet^4^.

The main pathological features of PD are the loss of the dopaminergic neurons in the substantia nigra region and the accumulation of α-synuclein-containing Lewy bodies in those neurons^2^. PD is defined as a complex disease with multiple underlying genetic and environmental factors^5^. Oxidative stress and mitochondrial dysfunction are the primary metabolic hallmarks of the degeneration of neurons in PD^6^. Gene expression profiling studies are widely used to investigate molecular mechanisms of the disease^7–9^. Numerous PD transcriptome datasets are available in databases like Gene Expression Omnibus (GEO)^10^.

Although clinical symptoms are widely utilized for the diagnosis of Parkinson’s disease, pathological indications of the disease appear at the molecular level several years before the clinical symptoms. Therefore, the investigation of preclinical biomarkers of PD is essential to diagnose the disease in the early stages, develop target-based therapeutical agents, and distinguish PD from other Parkinsonian diseases. Research on predicting molecular biomarkers commonly focuses on differential gene expression analysis to put forward gene biomarkers^11,12^. Yet, measuring gene-based biomarkers is hard compared to measuring metabolite-based biomarkers in terms of laboratory labor, cost, and time. Decrease in dopamine levels is a well-known PD metabolite biomarker^13^, and defining more metabolite-based biomarkers can be crucial in early diagnosis of PD before dopamine deficiency arises.

Biomarker prediction by integrative usage of computational models and omics data is an active research field^14,15^. TIMBR (Transcriptionally Inferred Metabolic Biomarker Response)^16^ is an algorithm that predicts metabolite biomarkers from the constraint-based analysis of genome-scale metabolic networks. TIMBR basically uses gene expression changes to calculate the network demands required for the secretion of metabolites, and compares them between two states. Before, it was successfully applied to predict early metabolite markers for toxicant-induced organ damages in rats^17–19^ and to analyze the changes in the secretion rates of metabolites for different experimental mouse models of PD^20^. Genome-scale metabolic models (GEMs) provide a mathematical platform for the integration of omics data for predicting condition specific metabolic behaviors of organisms. The publication of the first genome-scale metabolic network of Homo sapiens, Recon1^21^, made it possible to investigate human physiology, pathology, and pharmacology^22,23^. The unified human GEM (Human-GEM)^24^ is the last released human GEM. The human GEMs are commonly used for the systematic investigation of the metabolic mechanisms of diseases^25–27^, including brain-related diseases^28–30^.

In this study, thirteen PD related transcriptome datasets were mapped on Human-GEM by the TIMBR algorithm and its modified versions to provide a powerful computational biomarker prediction approach. Transcriptome datasets from the most affected brain region in PD, substantia nigra, were chosen to better capture PD-associated metabolic alterations. Metabolites predicted to have changed secretion profiles for the majority of the datasets were considered to be potential biomarkers with a meta-analysis approach. Metabolite and pathway-based metrics were used to compare the algorithms. A reaction on–off based modification of TIMBR algorithm introduced in this study, called TAMBOOR (TrAnscriptome-based Metabolite Biomarkers by On–Off Reactions), was found to be the most powerful approach. The metabolites predicted by TAMBOOR were suggested as candidate biomarkers for PD.

## Materials and methods

**Figure 1:**
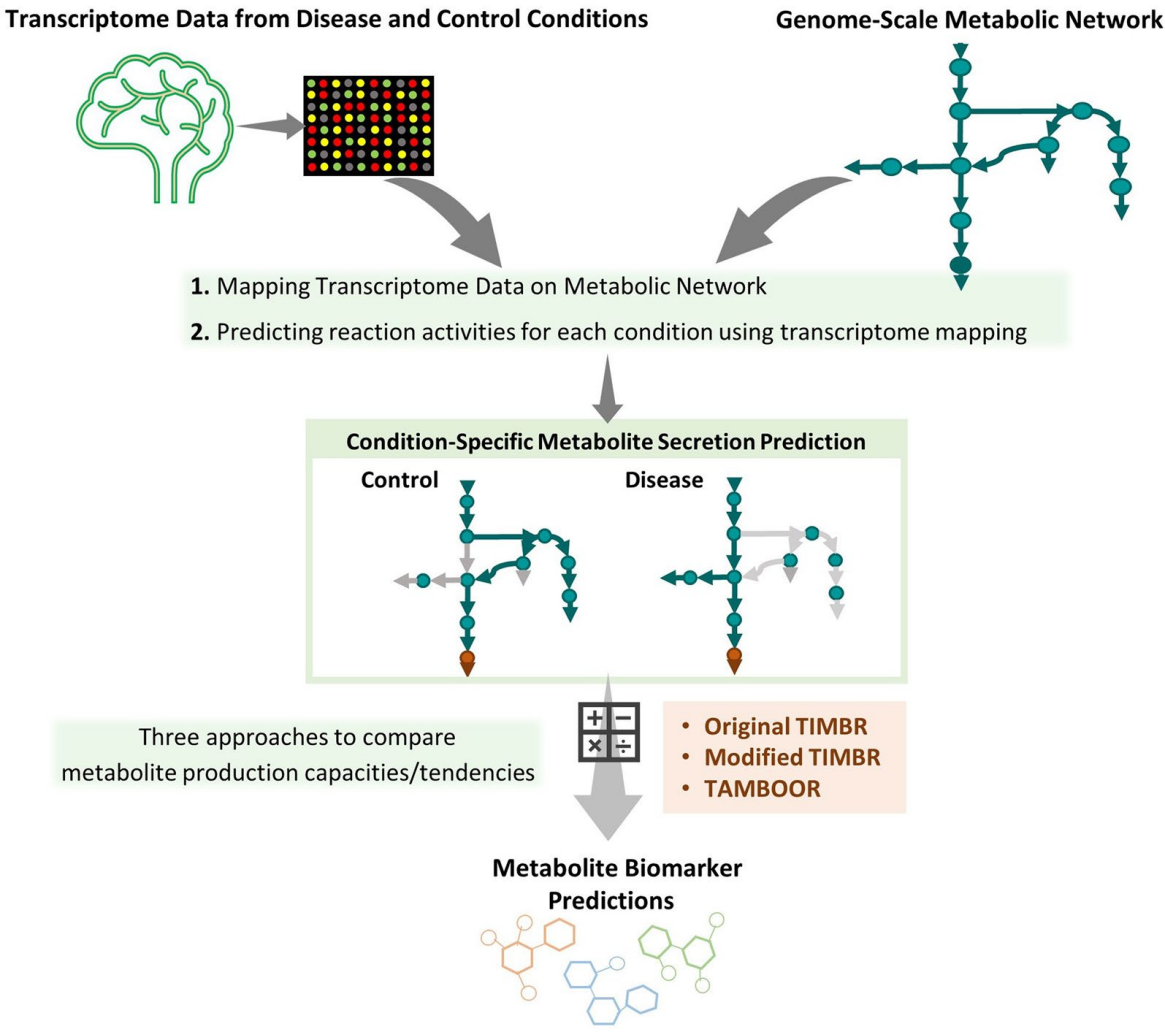
The basic pipeline followed in this study for predicting metabolite biomarkers by the integration of omics data and genome-scale metabolic networks.

The methodology of this study is summarized in Fig. 1.

### Human-GEM

The generic genome-scale metabolic model of *Homo sapiens* (Human-GEM)^24^ version 1.11.0 was used in this study. The model consists of 13,069 reactions, 3,067 genes and 8,366 metabolites. The simulations were performed in Ham’s medium, which consists of 44 metabolites (Supplementary Table S1)^24^, and three additional metabolites, which are taurine, ornithine and NH_3_. The uptakes of those metabolites were allowed in the simulations whereas the uptakes of all other metabolites in the model were blocked by setting the lower bounds of the rates through corresponding exchange reactions to zero. Maximum glucose and oxygen uptake rates were respectively constrained as 0.32 μmol/g/min and 1.76 μmol/g/min, the values from literature for the resting state brain metabolism^31,32^. Maximum uptake rates of fourteen amino acids (histidine, isoleucine, leucine, lysine, methionine, phenylalanine, threonine, tryptophan, valine, tyrosine, cysteine, arginine, ornithine and taurine), NH_3_ and other carbon source metabolites in Ham’s medium were constrained to be 1/10 of the glucose uptake rate, based on their reported relative rates^31^. As an additional constraint, the biomass production rate was constrained to be at least 0.0001 μmol/g/min to ensure that macromolecule synthesis is active in all conditions.

### Transcriptome data

Thirteen PD-related human transcriptome datasets presented in Table 1 were used to analyze changes in metabolite secretions in the substantia nigra region by using the biomarker prediction algorithms. Some of these datasets contain samples from different brain regions and/or different neurodegenerative diseases and expression profiles coming from distinct microarrays. Since the substantia nigra is the most affected brain region in PD, only post-mortem substantia nigra samples belonging to PD and control groups were considered. GSE8397 dataset includes expression profiles of lateral and medial substantia nigra regions separately. Therefore, 14 different comparisons from 13 datasets were evaluated in this study. The datasets include 112 control samples and 141 PD samples in total.

**Table 1:** Information about the PD transcriptome datasets used in this study. (C: Control, PD: Parkinson’s disease, LSN: Lateral substantia nigra, MSN: Medial substantia nigra).

Accession codes	Source	Technique and platform	Sample size	Reference
GSE7621	Substantia nigra	Microarray—Affymetrix Human Genome U133 Plus 2.0 Array	25 (9C–16PD)	^33^
GSE8397	Lateral and Medial Substantia nigra	Microarray—Affymetrix Human Genome U133A Array	LSN: 14 (5C–9PD) MSN: 25 (89C–15PD)	^34^
GSE20141	Substantia nigra	Microarray—Affymetrix Human Genome U133 Plus 2.0 Array	18 (8C–10PD)	^35^
GSE20163	Substantia nigra	Microarray—Affymetrix Human Genome U133A Array	17 (9C–8PD)	^35^
GSE20164	Substantia nigra	Microarray—Affymetrix Human Genome U133A Array	11 (5C–6PD)	^35^
GSE20292	Substantia nigra	Microarray—Affymetrix Human Genome U133A Array	29 (18C–11PD)	^7^
GSE20333	Substantia nigra	Microarray—Affymetrix Human HG-Focus Target Array	12 (6C–6PD)	–
GSE26927	Substantia nigra	Microarray—Illumina humanRef-8 v2.0 expression beadchip	20 (8C–12PD)	^36^
GSE54282	Substantia nigra	Microarray—Affymetrix Human Gene 1.0 ST Array	6 (3C–3PD)	^8^
GSE136666	Substantia nigra	RNAseq—Illumina HiSeq 2000	10 (5C–5PD)	^37^
GSE24378	Substantia nigra	Microarray—Affymetrix Human X3P Array	17 (9C–8PD)	^35^
GSE114517	Substantia nigra	RNAseq—Illumina NextSeq 500	27 (10C–17PD)	^38^
GSE49036	Substantia nigra	Microarray—Affymetrix Human Genome U133 Plus 2.0 Array	23 (8C–15PD)	^39^

The datasets were downloaded from Gene Expression Omnibus (https://www.ncbi.nlm.nih.gov/geo/) in non-normalized format. The normalizations were performed as detailed in our previous study^40^. Briefly, robust multi-chip average (RMA) and quantile normalization methods were applied for background correction and normalization of the microarray datasets whereas the RNA-seq datasets were normalized by the Trimmed Mean of M-values (TMM) method. Principal Component Analysis was applied to each normalized dataset to check for outliers^40^. Sample GSM506020 from dataset GSE20164, sample GSM208633 from dataset GSE8397, sample GSM663086 from dataset GSE26927, samples GSM509556 and GSM509557 from dataset GSE20333, samples GSM4054769 and GSM4054767 from dataset GSE136666, and samples GSM606624, GSM606625 and GSM606626 from dataset GSE20292 were determined as outliers and excluded from further analysis. GSE49036 contains PD samples including Braak stage information. Only the samples having Braak stage between 3 and 6 were assumed as PD samples.

### Biomarker prediction via TIMBR and alternative algorithms

**Figure 2:**
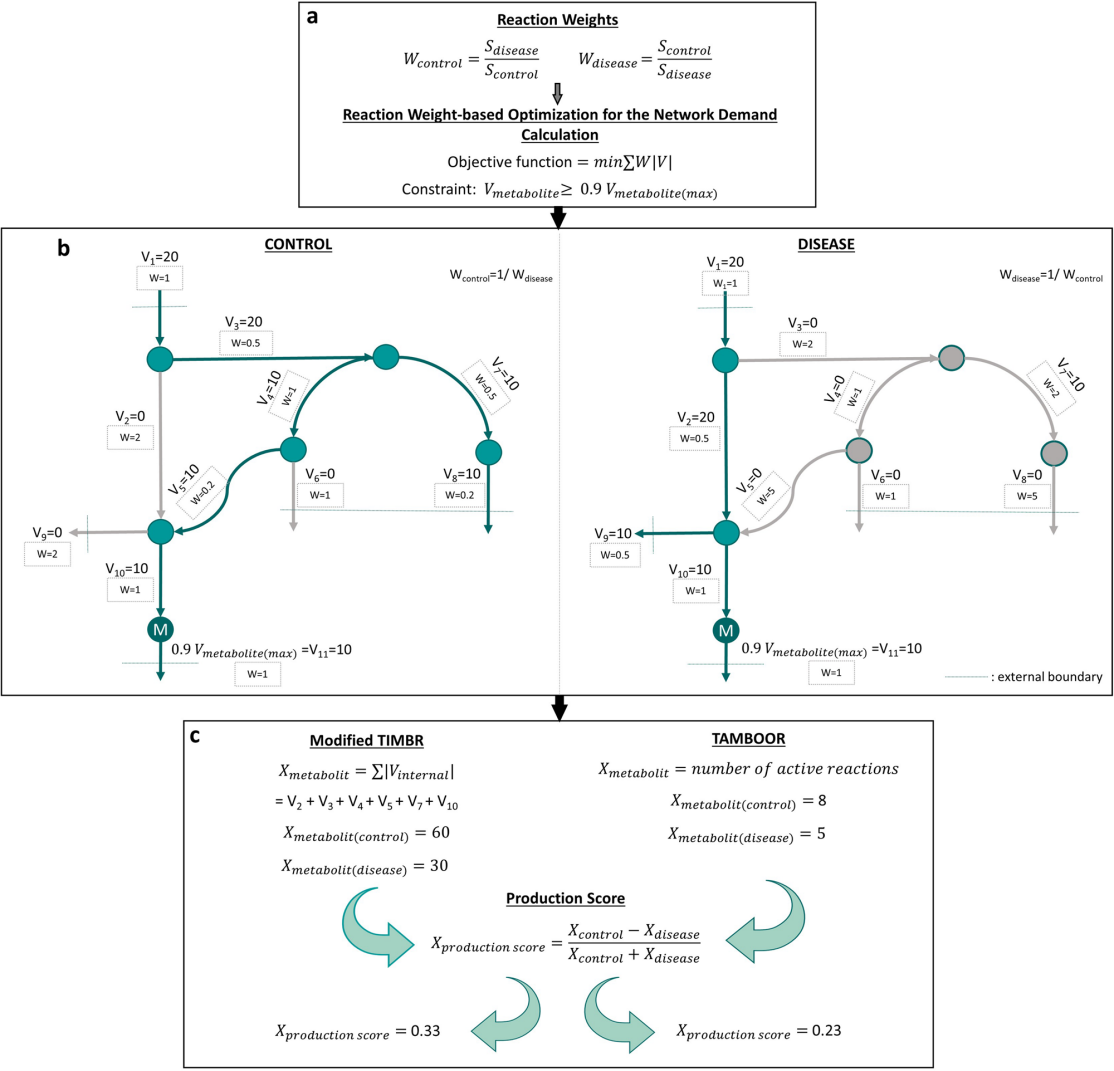
Modified TIMBR and TAMBOOR approaches for predicting metabolite biomarkers based on the gene expression changes. (**a**) Reaction weights are calculated as fold changes of transcriptome-based reaction scores obtained by mapping transcriptome data on the genome-scale metabolic model. The minimization of the weighted sum of fluxes is used as the objective function to predict flux distribution correlated with the upregulation/downregulation information. 90% of the maximum secretion flux through the related metabolite is used as a constraint in the optimization. (W: weight, S: reaction score, V: flux vector of reactions, V_metabolite_: the secretion rate of the metabolite of interest and X_metabolite_: the network demand) (**b**) Toy models to show how flux distributions differ based on the reaction score fold changes in control and disease states. The weight-based minimization strategy forces fluxes to pass through reactions with lower objective function coefficient values. (**c**) Network demand and production score calculations in modified TIMBR and TAMBOOR approaches. The summation of the internal fluxes is calculated as the network demand in the modified TIMBR whereas the number of active reactions is used to represent the network demand in TAMBOOR. Then, network demand differences between two conditions are used to calculate production scores.

The transcriptionally inferred metabolic biomarker response (TIMBR) algorithm^16^ was used here to predict biomarker potential of extracellular metabolites in PD patients. Briefly, transcriptome data from controls and PD patients were separately mapped on the human genome-scale metabolic network, Human-GEM, using GPR (gene-protein-reaction) rules defined in the model for each reaction. In this mapping, expression values of genes were assigned to reactions as follows: For the reactions controlled by a single enzyme, the gene expression value of the responsible gene was directly assigned to the reaction. The maximum of the expression values of responsible genes was assigned to the reactions controlled by isoenzymes, which are different enzymes catalyzing the same reaction independently. The minimum of the expression values was assigned to the reactions controlled by enzyme complexes, which consist of at least two enzymes that catalyze the reaction together. The expression values assigned to the reactions with this approach were referred to as reaction scores. Then, for each metabolite with a defined secretion reaction in the model, a linear-programming based optimization was performed to predict a flux distribution that satisfied a high secretion flux through the metabolite (Fig. 2). The TIMBR algorithm solves an optimization problem with the minimization of the weighted sum of fluxes, where the weights of the objective function are calculated as fold changes of transcriptome-based reaction scores from the GPR rules (control/disease for disease case, and disease/control for control case simulations). With this weight-based optimization strategy, reactions with the higher objective function coefficient values (lower expression values in the case of interest) were forced to take lower flux values. In this way, a flux vector is predicted such that it is correlated with the upregulation/downregulation information at mRNA level. The algorithm we implemented here includes some modifications to the original algorithm^16^. The modified parts of TIMBR were detailed in our previous study^20^.

In the TIMBR algorithm, the sum of weighted reaction fluxes is minimized (Eq. 1) by setting the lower boundary of the metabolite secretion reaction of interest to 90% of its maximum rate (Eq. 2). In this way, it is possible to calculate minimum network demand for the production of each metabolite. In this optimization, irreversible form of the metabolic model obtained by using *convertToIrreversible* function in the COBRA Toolbox^41^ was used to calculate flux values. The weighted network demand is calculated as a result of the optimization.

1 $$objective\, function=min\sum W|V|$$

2 $$V_{metabolite} \ge 0.9 V_{{metabolite\left( {\max } \right)}}$$

3 $${X}_{metabolite}=min\sum W|V|$$

In Eq. (1), W is the weight vector consisting of the reaction weights as fold changes from transcriptome data, and V is flux vector of reactions. In Eq. (2), V_metabolite_ is the secretion rate of the metabolite of interest. In Eq. (3), X_metabolite_ is the network demand. Based on these equations, network demands were calculated for both PD and control conditions separately for each metabolite. Human-GEM was used with the described constraints in the previous section to apply the TIMBR algorithm. Network demand differences between the two conditions were used to calculate production scores as in Eq. (4)^16^. The metabolites with a high production score were considered as biomarker metabolites with increased production potential in PD, whereas the metabolites with a low production score were accepted as metabolites with reduced secretion in PD.

4 $${X}_{production score}=\frac{{{X}_{control}-X}_{disease}}{{{X}_{control}+X}_{disease}}$$

The alternative versions of TIMBR considered here differs in terms of how network demand (X_metabolite_) is represented. We used two different alternatives here (Fig. 2). The first one, also applied in our previous study^20^, uses the sum of the internal fluxes as the network demand required for the production of the metabolite as shown in Eq. (5), where V_internal_ is the internal fluxes.

5 $${X}_{metabolite}=\sum |{ V}_{internal}|$$

Assuming a correlation between enzyme levels and fluxes, a lower value of the sum of fluxes in the disease condition compared to the control condition implies a lower total enzyme requirement for the high-level production of a metabolite in the disease condition (Eq. (2)). This can be interpreted as a higher tendency towards the production of the metabolite in the disease condition. The original TIMBR formulation (Eq. (3)), on the other hand, multiplies each flux with the transcriptome-based fold changes.

As a second alternative, X_metabolite_ was calculated based on the number of active reactions in the flux distributions predicted by the optimization defined in Eq. (1). Reactions with flux values higher than 10^–5^ were assumed to be “on” (active) reactions, while the others were assumed to be “off” (inactive). X_metabolite_ was calculated as shown in Eq. (6).

6 $${X}_{metabolite}=number\, of\, active\, reactions$$

In this alternative approach, we compared the path lengths for the high-level production of a metabolite in disease and control cases. A shorter path means a low number of enzymes. Thus, the shorter path in the disease state for the production of a given metabolite can be interpreted as an indication of higher production potential for that metabolite since its production is possible with a smaller number of enzymes (Fig. 2). The production score calculation and interpretation steps for this new modified version are the same as other versions of TIMBR as explained above. This reaction on–off based approach (Eq. (6)) led to the most promising results among the three versions (see results), and it was named as TrAnscriptome-based Metabolite Biomarkers by On–Off Reactions (TAMBOOR).

### Metabolite-based pathway enrichment analysis

Metabolite enrichment analysis was performed using the Metabolites Biological Role (MBROLE) 2.0 online tool^42^ (https://csbg.cnb.csic.es/mbrole2/) for metabolites whose secretion behavior were predicted to be changed in PD. A merged list consisting of metabolites whose secretion rates were predicted to be increased or decreased in at least 7 comparisons out of 14 comparisons for the substantia nigra region was utilized for the enrichment analysis. All metabolites analyzed in this study were given in Supplementary Information S1 with the corresponding number of times they were predicted as increased/decreased. The Kyoto Encyclopedia of Genes and Genomes (KEGG) pathway database^43^ library was selected to identify significantly affected metabolic pathways. The KEGG IDs of metabolites were used to apply the enrichment analysis based on the KEGG pathway database library in MBROLE.

## Results and discussion

In Human-GEM, 1036 metabolites can be secreted in silico based on our simulations on maximizing metabolite productions. For each of the fourteen PD-control comparisons, the production scores of 1036 secreted metabolites in the model were calculated with TIMBR and the two modified versions (Fig. 2) to predict potential increase or decrease in their secretion rates in the disease condition. Metabolites detected in the first 25th percentile of production score ranking were assumed to have a high probability of secretion in PD patients while metabolites detected in the last 25th percentile were considered to have a high probability of secretion in controls. These metabolites were defined as candidate biomarkers for the disease.

### Comparative evaluation of biomarker prediction approaches

Biomarker prediction powers of the three approaches (original TIMBR, modified TIMBR and TAMBOOR) were initially compared based on three metrics: the number of true predictions based on well-known PD metabolite biomarkers, the number of PD-related pathways in the metabolite enrichment analysis, and the number of unique metabolites included in the PD-related enriched pathways.

**Figure 3:**
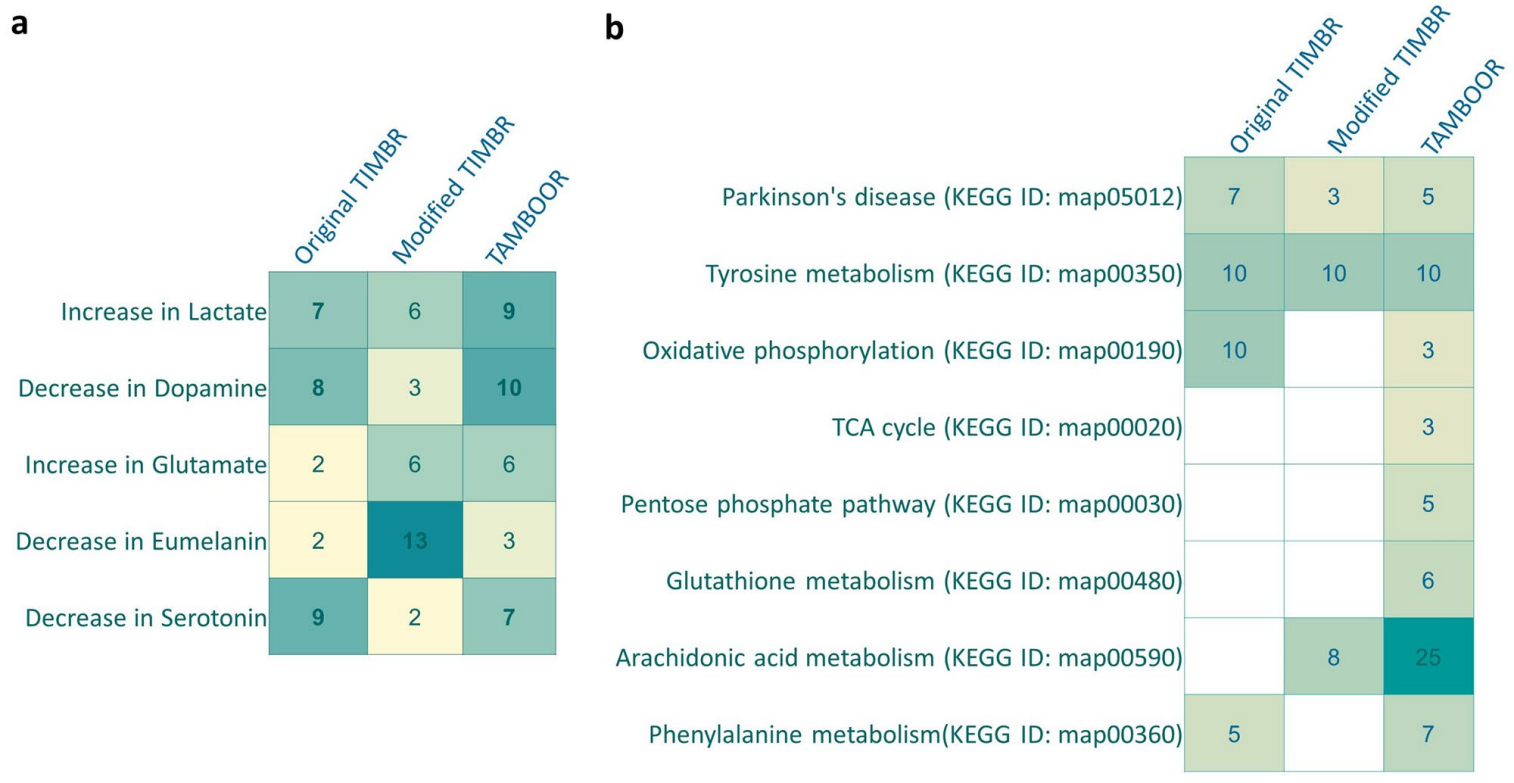
Predictions of the well-known PD-associated metabolite secretion changes and PD-associated pathways by three algorithms. (**a**) The well-known PD-associated metabolite secretion changes and the number of comparisons in which these metabolite secretion changes were correctly predicted. (**b**) Significantly enriched PD-associated pathways and the number of related metabolites whose secretion rates were predicted to be altered by the given approach.

The first metric is the number of true predictions, which is determined based on the number of comparisons in which the well-known PD-related changes in metabolite secretion rates were truly predicted. These changes are increase in lactate^44^, glutamate^45,46^ and decrease in dopamine^47^, eumelanin^2^ and serotonin secretions^48^. The number of comparisons in which the well-known PD-related metabolite production changes were correctly predicted was given in Fig. 3a for each algorithm. Metabolites predicted as biomarkers in at least 7 comparisons out of 14 comparisons were assumed to have a high potential for being biomarkers and considered in further analysis. TIMBR and TAMBOOR could predict increase in lactate secretion and decrease in dopamine and serotonin secretions successfully whereas the modified TIMBR approach could predict only the decrease in eumelanin level.

The second and third metrics are related to enriched pathways. In order to compare metabolite enrichment analysis results, seven PD-related pathways, which are Parkinson’s disease, tyrosine metabolism, oxidative phosphorylation, TCA cycle, pentose phosphate pathway, glutathione metabolism and phenylalanine metabolism, were selected based on KEGG pathway annotation. Metabolite biomarker lists predicted by each algorithm were subjected to enrichment analysis to identify if they are commonly involved in these PD-related pathways. Significantly enriched PD-associated pathways were presented in Fig. 3b, with the number of pathway metabolites whose secretion rates were predicted to be altered. All of the seven PD-related pathways were found to be significantly enriched with the metabolites captured by the TAMBOOR approach. Parkinson’s disease, tyrosine metabolism, oxidative phosphorylation and phenylalanine metabolism terms were enriched based on the original TIMBR predictions. Only Parkinson’s disease and tyrosine metabolism terms were enriched when the metabolite enrichment analysis was applied to the metabolites predicted by the modified TIMBR. Different pathways identified to be enriched can be associated with the same altered metabolite. The main purpose of the biomarker prediction approaches applied here is to predict as many reliable metabolite biomarkers as possible. Therefore, the number of unique metabolites included in all of the significantly enriched PD-related pathways is also a critical metric for metabolite-level biomarker predictions, and it was used as the third metric in our analysis.

For a further examination of the predictive power of the three approaches, metabolites predicted as high-potential biomarkers by each approach were searched in the literature to determine whether the predicted changes are compatible with experimental studies. The predicted high-potential biomarkers supported by at least two experimental studies were assumed to be high-confidence biomarkers. A total of 25 metabolite-level changes were identified as high-confidence biomarkers, as listed in Table 2. The list of high-confidence biomarkers and the list of well-known PD-associated metabolite level changes (5 metabolites in Fig. 3a) add up to 30 metabolites in total, which were compared with the predicted metabolites by the three approaches to calculate statistical metrics: precision and recall. The number of predictions, true positive values, and calculated statistical metrics for each approach are given in Table 3. Precision was used to get information about how many positive predictions overlap with the known positives (Fig. 3a and Table 2) whereas recall was used to get information about how many known positives could be correctly predicted. The calculated recall values show that TAMBOOR can predict more than half of the known positives.

**Table 2:** The high-confidence metabolite level changes for PD compiled from the literature, with references of the supporting studies.

Metabolites (Increased)	Detected by	Supporting references
Quinolinate	Original TIMBR	^49,50^
Kynurenine	Original TIMBR	^51,52^
Fe3 +	Original TIMBR	^53,54^
Hydroxide	Original TIMBR	^55,56^
Prostaglandins	Original TIMBR	^57,58^
Salsolinol	TAMBOOR	^59,60^
1,2-dehydrosalsolinol	TAMBOOR	^61,62^
Acetone	TAMBOOR	^63,64^
Vanil-Lactate	TAMBOOR	^65,66^
Formate	TAMBOOR	^63,67^
Urea	TAMBOOR	^68,69^
(R)-mevalonate	TAMBOOR	^70,71^
Biliverdin	TAMBOOR	^69,72^
4-methyl-2-oxopentanoate	Original TIMBR & TAMBOOR	^73,74^
**Metabolites (Decreased)**	**Detected by**	
ApoA1 (Apolipoprotein A1)	Original TIMBR	^75,76^
5-hydroxyindoleacetate	Original TIMBR	^77,78^
Lanosterol	Original TIMBR	^79,80^
3-methoxytyramine	Original TIMBR	^81,82^
Lysine	Modified TIMBR	^83,84^
GSH (Glutathione)	Modified TIMBR	^85,86^
Calcitriol (Vitamin D)	TAMBOOR	^87,88^
Nicotinamide	TAMBOOR	^89,90^
Carnosine	TAMBOOR	^91,92^
Albumin	Original TIMBR & TAMBOOR	^93,94^
Thiamin-P	ALL	^95,96^

**Table 3:** The number of predictions, true positive values, and calculated statistical metrics for each biomarker prediction approach considered in the study.

	Original TIMBR	Modified TIMBR	TAMBOOR
Number of predicted biomarkers (increased secretion)	184	178	118
Number of predicted biomarkers (decreased secretion)	185	121	156
Total number of predicted biomarkers	369	299	273
Number of high confidence markers in predictions	12	3	14
Number of well known markers in predictions	3	1	3
Total True Positives	15	4	17
Precision = TP/(TP + FP)	0.041	0.013	0.062
Recall = TP/(TP* + FN**)	0.484	0.133	0.567

*TP* true positive, *FP* false positive, *FN* false negative.

*TP: Number of high confidence markers in predictions + Number of well known markers in predictions.

**FN: (Number of high confidence markers + Number of well known markers = 30)—TP.

**Figure 4:**
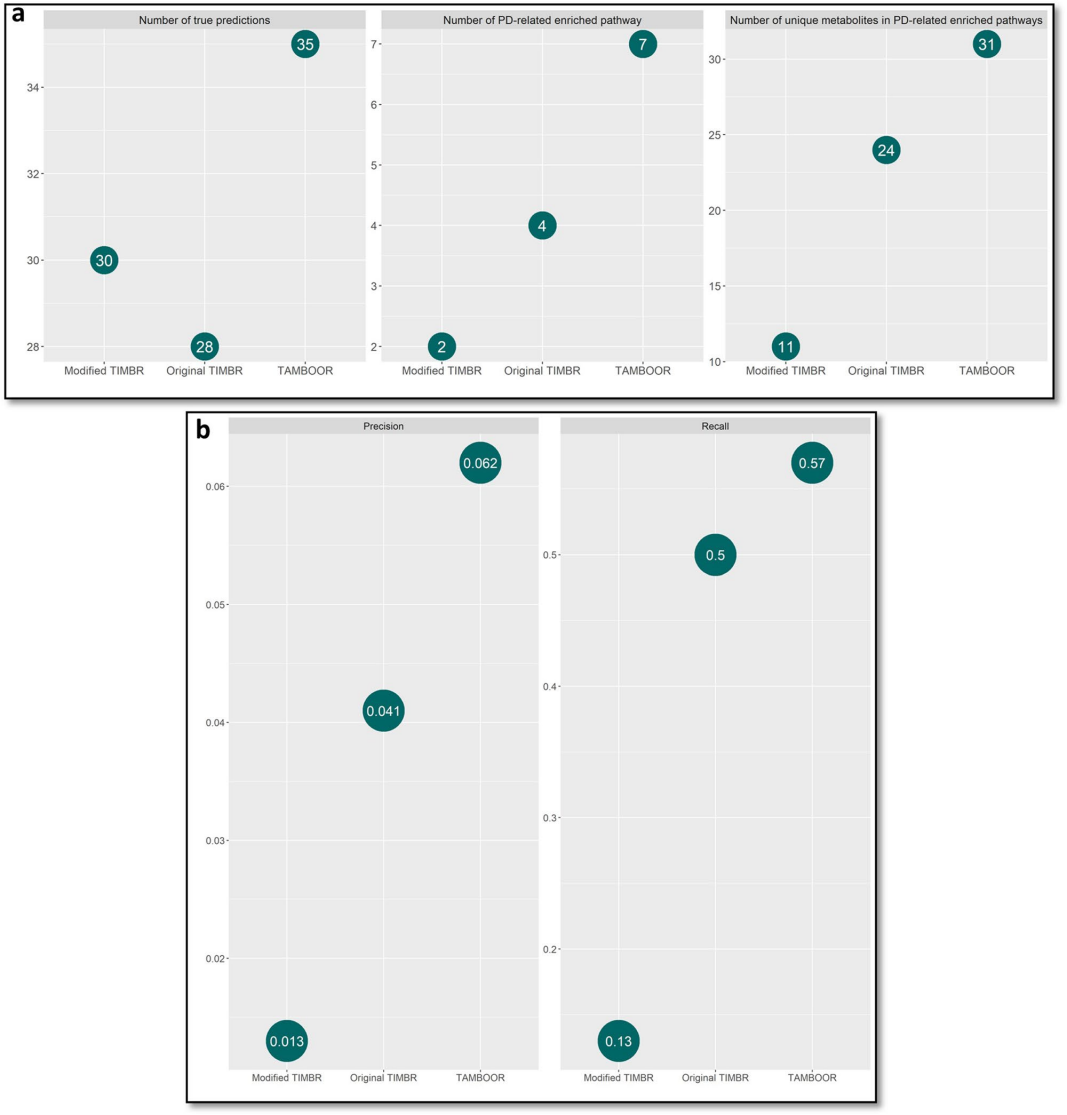
Metrics for the comparison of the biomarker prediction approaches. (**a**) Statistical metrics. (**b**) Metrics based on well-known metabolic changes.

The results of the metrics used to compare different biomarker prediction approaches were represented in Fig. 4 for each algorithm. The highest number of true predictions, the highest number of PD-related enriched pathways, the highest number of unique metabolites included in the PD-related enriched pathways, and the highest precision and recall values were obtained by TAMBOOR. Based on these metrics, TAMBOOR was suggested as the most powerful approach to predict metabolite biomarkers for PD.

The performance of computational prediction approaches can differ based on the properties of the used model and data. In our previous work^20^, the changes in the metabolite secretion rates for different experimental mouse models of PD were successfully predicted with the modified TIMBR approach while it did not perform well in this study. Whereas a brain-specific metabolic network model of mouse (iBrain674-Mm) with 992 reactions controlled by 674 genes was used in that study for the production potentials of 44 secreted metabolites, the model used in the current study, Human-GEM, enabled the screening of 1036 secreted metabolites. The logic of the modified TIMBR relies on the sum of internal fluxes, and Human-GEM consists of numerous metabolite transport reactions between cell compartments. The sum of the fluxes of those transport reactions may have dominated the overall sum for the modified TIMBR approach, obscuring the differences between controls and patients in a larger metabolic network model.

### Altered metabolite productions in PD based on TAMBOOR predictions

Extracellular levels of 118 metabolites were predicted to be increased in at least 7 comparisons with TAMBOOR, whereas levels of 156 metabolites were predicted to be decreased in at least 7 comparisons out of 14 comparisons (Supplementary Information S1). The decrease in dopamine production, the well-known metabolic change in PD, was detected in 10 of 14 comparisons. Also, the level of dopamine precursors, tyrosine and L-dopa, were predicted to be decreased for 9 and 7 comparisons respectively. In addition, an increase in lactate production and a decrease in serotonin level, other well-known changes in PD, were detected in 9 and 7 comparisons respectively.

TAMBOOR predicted 14 out of 25 high-confidence biomarkers, with 11 of them predicted only by TAMBOOR. Three of them are salsolinol, 1,2-dehydrosalsolinol, and 4-methyl-2-oxopentanoate (ketoleucine), whose productions were predicted to be increased in 10 comparisons. Salsolinol is a dopamine-derived compound. Its structure is very similar to 1-methyl-4-phenyl-1,2,3,6-tetrahydropyridine (MPTP), a compound used to create experimental PD models. It can be described as a neurotoxin that contributes to the loss of dopaminergic neurons in PD^60^. Increased salsolinol levels were detected in the cerebrospinal fluid of PD patients with dementia^97,98^. Besides, the increase in salsolinol levels was associated with visual hallucinations in PD^59^. 4-methyl-2-oxopentanoate, another metabolite predicted by TAMBOOR, inhibits mitochondrial complex I, and its levels were reported to be increased in the plasma and cerebrospinal fluid (CSF) of PD patients in metabolome studies^73,74^. The productions of acetone, formate, urea and biliverdin were also predicted to be increased in 7–8 comparisons. Elevated serum levels of acetone and formate were reported in PD by a metabolome study^63^. Both metabolites have roles in mitochondrial functions, which are affected in PD. Urea levels have also been reported as elevated for different brain regions in PD patients with dementia^68^. It was demonstrated that increased serum level of biliverdin is correlated with reactive oxygen species (ROS) level and disease severity^69^. Productions of carnosine, nicotinamide and calcitriol (vitamin D) were predicted to be decreased in 9, 8 and 7 comparisons, respectively. A reduced level of carnosine was suggested as a diagnostic marker for AD^91^. Carnosine administration was tested as a treatment strategy in a mouse model of PD, and the results showed that daily carnosine intake can reduce PD symptom progression^92^. It was shown that experimentally created nicotinamide deficiency caused dopamine reduction, cell loss, and consequently parkinsonism^90^. It was also reported that elevated levels of nicotinamide N-methyltransferase reduces nicotinamide level and consequently Complex I activity in idiopathic PD^89^. Vitamin D deficiency was observed in PD patients and calcitriol, which is the active form of vitamin D, was shown to be neuroprotective for PD animal models^87,88^.

In addition to the well-known metabolite level changes and the high-confidence biomarker list, several other metabolites predicted by TAMBOOR were associated with Parkinson's metabolism and neurodegeneration, and, they can be considered as candidate biomarkers. The increase in the production of hexanoic acid (hexanoylcarnitine), a medium-chain acylcarnitine, was predicted in 11 of 14 comparisons. In an experimental study^99^, the contribution of hexanoylcarnitine accumulation to oxidative stress in rat brains was demonstrated. Hexanoylcarnitine accumulation debilitates the antioxidant defense of the brain by decreasing glutathione levels. Hexanoylcarnitine increase has also been reported as an age-related change in rat brain medial prefrontal cortex^100^. Consequently, hexanoic acid/hexanoylcarnitine can be considered as a candidate biomarker for PD. Acetyl-threonine production was predicted to be increased in 10 comparisons. In a study, almost a two-fold change in acetyl-threonine level was detected between control cells and cells exposed to 100 nM rotenone, a chemical used to create experimental PD models^101^. It is also known that acetyl-threonine has roles in neuronal growth and different neuronal functions^102^. Therefore, a change in its level is supposed to be related to neurodegenerative diseases. The productions of heparan sulfate proteoglycans, glycan, glycochenodeoxycholate and leukotriene D4 (LTD4) were predicted to be increased in 9 comparisons in this study. Cell surface heparan sulfate proteoglycans promote neuronal internalization of α-synuclein and also other amyloid forming proteins in neurodegenerative diseases by contributing to neuronal binding and uptake of protein fibrils^103^. Therefore, the predicted increase in heparan sulfate proteoglycans production is in line with the main pathology of PD, α-synuclein aggregation. Glycan was suggested as a potential biomarker for neurological diseases^104^. An elevated level of glycochenodeoxycholate was reported for mild cognitive impairment (MCI) and Alzheimer's disease (AD)^105^. Leukotriene D4 is a subgroup of cysteinyl leukotrienes produced by  the oxidation of arachidonic acid. Cysteinyl leukotrienes, inflammatory lipid mediators, have been associated with neurodegenerative diseases, including PD^106^. An increase in xanthurenate level was reported in serotonin deficiency^107^. The decrease in serotonin levels is one of the well-known metabolic changes in PD. Therefore, predicting increased xanthurenate production in 8 comparisons is consistent with the experimental studies.

Production of histamine was predicted to be decreased in 11 comparisons. Histamine has important roles in modulating striatal synaptic transmission and behavior^108^. Since striatum is one of the most affected brain regions in PD, reduced histamine secretion in PD should be confirmed in further studies. Production of chloride was predicted to be decreased in 10 comparisons. Chloride is an electrolyte that has an essential role in controlling neuronal excitability. Low levels of chloride have been associated with dyskinesia in PD patients^109^. Cysteinyl-glycine and arginine productions were predicted to be decreased in 8 comparisons. Reduced cysteinyl-glycine was detected as a marker for levodopa-induced oxidative stress in PD patients^110^. Arginine level in the CSF of PD patients was reported to be low compared to the controls^84^.

Metabolites that were predicted to have altered secretion behavior in PD were searched on Human Metabolome Database (HMDB)^111^ to check if they were previously associated with any disease. Many of them were found to be linked to colorectal cancer in different studies. In recent years, impairments in the gut and neurological disorders have been closely associated with the gut-brain axis theory. It has been known that gut microbiota affects both PD and colorectal cancer (CRC) pathogenesis. Different studies have reported that PD patients have a significantly decreased risk for CRC^112,113^.

Most of our predictions are compatible with the experimental evidence in the literature. However, there are also some predictions conflicting with literature reports. For instance, a decline was reported for most of the amino acids, including lysine and taurine, in PD^84,114^. Contrarily, lysine and taurine productions were predicted to be increased by TAMBOOR. Most of the metabolites whose levels were predicted to be decreased by TAMBOOR are the metabolites of arachidonic acid metabolism, such as prostaglandins, lipoxins, hydroxyeicosatetraenoic acids (HETEs), and thromboxane A2. We could not link all of those alterations directly to PD based on literature survey. However, it was reported that the daily intake of arachidonate increases the PD risk^115^. Also, it was suggested that prostaglandins promote protein aggregation in PD^57^. Therefore, a decrease in the levels of arachidonic acid metabolism-related metabolites is not expected. Fe3 + production was predicted to be decreased for 8 comparisons, although elevated iron levels were associated with PD^116^.

Most of the TAMBOOR-based predictions with strong evidence could not be predicted by the original and modified TIMBR approaches. Decreased dopamine and serotonin production and increased lactate production capacities could not be predicted by the modified TIMBR. Decreased production capacity of dopamine precursors, tyrosine and L-dopa, was only detected by TAMBOOR. Modified TIMBR and original TIMBR, on the other hand, wrongly predicted increased production of L-dopa. Vitamin D deficiency, nicotinamide deficiency, reduced carnosine level, which is previously suggested as a diagnostic biomarker for neurodegenerative diseases, and elevated urea and salsolinol levels, which are previously detected as a metabolic change in neurodegenerative diseases including PD, were also only predicted by TAMBOOR. On the other hand, there are also some high-confidence metabolite level changes having strong literature evidence such as an increase in hydroxide level, and a decrease in glutathione and Apolipoprotein A1 levels, which could not be captured by TAMBOOR, but correctly predicted by the other algorithms.

GEM-based prediction algorithms like TIMBR and TAMBOOR have some limitations. For example, they assume that reaction activities are directly correlated with the expression levels of genes coding for the enzymes that catalyze the reactions. Another assumption is the scoring of the secretion tendency of a metabolite based on the number of active reactions or the weighted sum of fluxes through active reactions. Although these assumptions are biologically relevant, they cannot be exactly true for all cases. Still, it is important to develop a GEM-based metabolite biomarker prediction algorithm with a high predictive power to pave the way for novel PD biomarkers that can be useful for early diagnosis. The most common molecular-level biomarker prediction strategy in literature is discovering gene biomarkers using differential gene expression analysis. However, TIMBR and TAMBOOR predict metabolite biomarkers, which are easier to measure compared to gene biomarkers by taking samples from blood or CSF.

### Pathway-based changes in PD based on TAMBOOR predictions

Functional enrichment analysis was applied to the metabolites predicted to have altered secretion by TAMBOOR to identify metabolic pathways significantly enriched with those metabolites. Many PD-related and brain function-related terms were detected as significantly enriched (The False Discovery Rate (FDR) < 0.05). The list of significantly enriched pathways is given in Table 4 (All enrichment results are available in Supplementary Information S2). The comprehensive literature survey presented in this section shows that the majority of the enriched terms/pathways are associated with PD.

**Table 4:** Significantly enriched pathways (FDR < 0.05) based on the enrichment analysis applied to the metabolites predicted to have altered secretion by TAMBOOR.

Enriched KEGG pathways	*p*-value	FDR correction
Arachidonic acid metabolism	3.62 × 10^–21^	3.40 × 10^–19^
Metabolic pathways	3.64 × 10^–12^	1.71 × 10^–10^
Neuroactive ligand-receptor interaction	9.07 × 10^–8^	2.84 × 10^–6^
Asthma	5.25 × 10^–6^	1.23 × 10^–4^
Vascular smooth muscle contraction	1.13 × 10^–4^	2.13 × 10^–3^
Tyrosine metabolism	1.44 × 10^–4^	2.26 × 10^–3^
Fc epsilon RI signaling pathway	2.98 × 10^–4^	4.00 × 10^–3^
Phenylalanine metabolism	6.12 × 10^–4^	6.40 × 10^–3^
Parkinson's disease	6.13 × 10^–4^	6.40 × 10^–3^
Oxidative phosphorylation	1.45 × 10^–3^	1.36 × 10^–2^
Valine, leucine and isoleucine biosynthesis	1.83 × 10^–3^	1.56 × 10^–2^
Cysteine and methionine metabolism	2.03 × 10^–3^	1.59 × 10^–2^
ABC transporters	2.36 × 10^–3^	1.70 × 10^–2^
Pentose phosphate pathway	3.37 × 10^–3^	2.27 × 10^–2^
Gap junction	4.61 × 10^–3^	2.61 × 10^–2^
Riboflavin metabolism	4.19 × 10^–3^	2.61 × 10^–2^
Glycine, serine and threonine metabolism	4.71 × 10^–3^	2.61 × 10^–2^
Glutathione metabolism	7.20 × 10^–3^	3.56 × 10^–2^
Alanine, aspartate and glutamate metabolism	6.90 × 10^–3^	3.56 × 10^–2^
Thiamine metabolism	9.23 × 10^–3^	4.34 × 10^–2^
Epithelial cell signaling in Helicobacter pylori infection	9.90 × 10^–3^	4.43 × 10^–2^
Pantothenate and CoA biosynthesis	1.06 × 10^–2^	4.52 × 10^–2^

Phenylalanine metabolism, tyrosine metabolism, glutathione metabolism, and, arachidonic acid metabolism terms were listed as significantly enriched pathways in Table 4. Phenylalanine and tyrosine are precursors for the synthesis of catecholamines, including dopamine synthesis. Besides, dopaminergic neuron loss is one of the main pathological features of PD^65^. Hence, significant alterations in phenylalanine, tyrosine, and catecholamine metabolisms are expected in PD. Glutathione acts as an antioxidant and a redox regulator in brain metabolism. Glutathione level reduction is known as the first sign of oxidative stress during PD progression^117^. Therefore, affected glutathione metabolism is also one of the expected results for PD. Intermediate products of arachidonic acid metabolism have essential roles in the central nervous system, such as neurotransmitter transfer, synaptic signaling, and neuronal firing. Altered arachidonic acid metabolism was associated with different brain-related disorders, including PD^118^. Studies have reported that arachidonic acid consumption increases the risk of PD^115^ and suggested enzymes in the arachidonic acid metabolism as drug targets^119^.

Mitochondrial dysfunctions in energy metabolism and oxidative stress are important factors in PD pathogenesis^6^. The terms oxidative phosphorylation, pentose phosphate pathway, and ATP-binding cassette transporters, presented as significantly enriched pathways in Table 4, are also directly related to energy metabolism and oxidative stress. ATP-binding cassette (ABC) transporters are highly active in the brain, and their roles in some neurodegeneration-related processes were reported^120^. Riboflavin is an essential B vitamin in the nervous system because of its role in myelin synthesis. The oxidative state impairments related to riboflavin deficiency have been reported. Riboflavin intake was tested as a treatment for PD and other neurodegenerative diseases^121^. Also, thiamine is another type of vitamin B whose deficiency is related to PD^95^. Both riboflavin and thiamine metabolisms were identified in the enrichment analysis (Table 4).

PD is typically characterized by tremors and muscle stiffness. The term vascular smooth muscle contraction in the enrichment analysis results (Table 4) is directly related to this symptom. Vascular smooth muscle cell dysfunction was considered as a crucial marker to interpret alterations in vascular function at the onset and progression of neurodegenerative diseases, including Parkinson’s disease^122^. Also, the relationship between vascular smooth muscle contraction and PD was demonstrated in another enrichment-based analysis^123^. Pantothenate and CoA biosynthesis is also one of the significantly enriched pathways. Variations in pantothenate kinase (PKAN) causes interruptions in coenzyme A (CoA) biosynthesis and consequent iron accumulation in the brain. This phenomenon is related to many neurodegenerative clinical symptoms, including rigidity, loss of ambulation, and cognitive and visual impairment^124^.

Alanine, aspartate and glutamate metabolism was found to be enriched in the enrichment analysis results as shown in Table 4. The relation between the changes in glutamate metabolism and neuronal metabolic dysfunctions is known to play an important role in the pathophysiology of PD^125^. Glutamate is an excitatory transmitter whose excess levels are neurotoxic. Mechanisms for reducing glutamate toxicity, like removing excessive synaptic glutamate by excitatory amino acid transporter (EATT), are known to be affected in PD^45^. Glutamate aspartate transporter is an important EATT for the synaptic reuptake of glutamate^126^. Thus, it can be concluded that the enriched alanine, aspartate and glutamate metabolism term is directly related to altered glutamate toxicity and transport mechanism in PD. Epithelial cell signaling in Helicobacter pylori infection was reported as an enriched pathway in Table 4. It was previously reported that bacterial overgrowth and *Helicobacter pylori* infection in the small intestine affected motor fluctuations by interfering with the absorption of antiparkinsonian drugs^127^. The enriched term, epithelial cell signaling in Helicobacter pylori infection, is most probably related to this phenomenon. Asthma is another significantly enriched term in the enrichment analysis. It was also previously reported that asthma patients are at higher risk of developing PD in later life^128^.

## Conclusion

The constraint-based modelling approaches are effectively used to investigate disease metabolism by integrating omics data. Here, we compared the prediction capabilities of the original TIMBR, the modified TIMBR and TAMBOOR approaches to identify potential metabolite biomarkers for Parkinson's disease using 13 different transcriptome datasets. A high-confidence metabolite biomarker list for Parkinson's disease was compiled by a literature survey for the predicted metabolite level changes by different algorithms. Different metrics covering the prediction of well-known and high-confidence metabolite level changes and the prediction of metabolites belonging to PD-related metabolic pathways were used to measure biomarker prediction abilities of the three approaches. TAMBOOR reached the highest scores for these metrics.

Metabolites predicted to have altered secretion rates by TAMBOOR and enriched pathways corresponding to these metabolites are mainly related to PD-related metabolisms. Majority of the predicted alterations are consistent with the literature. However, an experimental study that reports secretion rates of all of the metabolites predicted in this study is not available for healthy and disease subjects. Well-designed comprehensive metabolome analyses will be valuable for a complete validation of our results.

The metabolites whose secretion behavior were predicted to be changed by TAMBOOR are candidate biomarkers. Moreover, the metabolites predicted to have increased production capacity can be potential drug targets while the metabolites predicted to have decreased production capacity have the potential to be used as supplementary therapeutics in PD. Those potentials should be validated with experimental studies to identify novel diagnostic biomarkers and treatment strategies for PD.

### Supplementary Information

Supplementary Information 1.

Supplementary Information 2.

Supplementary Information 3.

## Data Availability

The normalized datasets are available from the corresponding author on reasonable request.
