# Supplementary material for: Transcriptome-based biomarker prediction for Parkinson’s disease using genome-scale metabolic modeling
Source: Sci Rep. 2024 Jan 5;14:585. doi: 10.1038/s41598-023-51034-y (PMC10770157; doi:10.1038/s41598-023-51034-y)
Supplement: Supplementary file 3 — Supplementary Information 3. [file 41598_2023_51034_MOESM3_ESM.pdf]

Transcriptome-Based Biomarker Prediction for Parkinson's Disease Using Genome-Scale Metabolic Modeling

Ecehan Abdik[1], Tunahan Çakır[1]*

[1]Department of Bioengineering, Gebze Technical University, Kocaeli, Turkey.

*Corresponding Author

E-mail: tcakir@gtu.edu.tr

Table S1. Metabolites and their maximum uptake rates used in constraint-based modeling simulations

Metabolites	Maximum Uptake Rates (µmol/g/min)
alanine	0.032
alpha-tocopherol	0.032
aquacob(III)alamin	0.032
arginine	0.032
asparagine	0.032
aspartate	0.032
biotin	0.032
choline	0.032
cysteine	0.032
Fe2+	1000
folate	0.032
gamma-tocopherol	0.032
glucose	0.32
glutamate	0.032
glutamine	0.032
glycine	0.032
H2O	1000
histidine	0.032
hypoxanthine	0.032
inositol	0.032
isoleucine	0.032
leucine	0.032
linoleate	0.032
linolenate	0.032
lipoic acid	0.032
lysine	0.032
methionine	0.032
NH3	0.032
nicotinamide	0.032
O2	1.76
ornithnine	0.032
pantothenate	0.032
phenylalanine	0.032
Pi	1000
proline	0.032
pyridoxine	0.032

retinoate	0.032
riboflavin	0.032
serine	0.032
sulfate	1000
taurine	0.032
thiamin	0.032
threonine	0.032
thymidine	0.032
tryptophan	0.032
tyrosine	0.032
valine	0.032
